# Proteomic Identification of IL4I1 as a Therapeutic Target in P53-Mutant Endometrial Cancer

**DOI:** 10.3390/cancers17182986

**Published:** 2025-09-12

**Authors:** Hu Li, Ruonan Zhang, Tiantian Sui, Kai Wang, Yiran Li, Xuezhen Luo, Qizhi He

**Affiliations:** 1 Shanghai Key Laboratory of Maternal Fetal Medicine, Shanghai Institute of Maternal-Fetal Medicine and Gynecologic Oncology, Shanghai First Maternity and Infant Hospital, Tongji University School of Medicine, Shanghai 200092, China; 2511148@tongji.edu.cn (H.L.);; 2 Department of Obstetrics and Gynecology, Peking University People’s Hospital, No. 11, Xizhimen South Street, Xicheng District, Beijing 100044, China; 3 Clinical and Translational Research Center, Shanghai First Maternity and Infant Hospital, Tongji University School of Medicine, Shanghai 200092, China; 4 Department of Gynecology, Obstetrics and Gynecology Hospital of Fudan University, Shanghai 200011, China; 5 Department of Pathology, Shanghai First Maternity and Infant Hospital, Tongji University School of Medicine, Tongji University, Shanghai 200092, China

**Keywords:** endometrial cancer (EC), proteomics, bioinformatics, biomarker, immunohistochemistry

## Abstract

**Background**: Endometrial cancer (EC) is one of the most common gynecologic malignancies, with a rising incidence worldwide. The Cancer Genome Atlas (TCGA) classifies EC into four molecular subtypes, among which the high-copy number subtype is characterized by TP53 mutations and associated with poor prognosis. However, this subtype remains understudied. IL4I1, an immunoregulatory enzyme implicated in various cancers, has emerged as a potential biomarker for tumor progression. This study aimed to explore IL4I1 as a prognostic marker in P53-mutant EC and to identify its potential as a therapeutic target. **Methods**: We retrospectively analyzed clinical data from 495 EC patients and selected 33 P53-mutant cases using Sanger sequencing and immunohistochemistry. Tumor tissues were collected via laser capture microdissection, stratified by five-year survival outcomes. IL4I1 expression was assessed through 4D label-free proteomics, immunohistochemistry, and Western blotting. TCGA data were used to validate expression patterns and prognostic significance. Functional analyses were performed using IL4I1-knockout P53-mutant EC cell lines generated via CRISPR/Cas9, followed by phenotypic assays and xenograft models. **Results**: IL4I1 was significantly upregulated in deceased patients and correlated with immune microenvironment alterations in TCGA data. Knockout of IL4I1 inhibited proliferation, migration, and invasion in vitro and tumor growth in vivo. **Conclusions**: IL4I1 is a key player in the aggressiveness of P53-mutant EC. It holds promise as a prognostic biomarker and may serve as a novel target for precision therapies in this high-risk EC subtype.

## Simple Summary

Endometrial cancer is one of the most common cancers affecting women. Among its types, those with changes in a gene called P53 tend to be more aggressive and harder to treat. Current treatment options are often not effective for these patients, and new strategies are urgently needed. In this study, we aimed to find new treatment targets for this aggressive form of cancer. Using advanced protein analysis techniques, we discovered that a molecule called IL4I1 is much higher in these tumors than in normal tissue. We then showed that this molecule helps the cancer grow and avoid the body’s immune system. When we blocked this molecule in laboratory experiments and animal models, tumor growth slowed down, and immune response improved. These findings suggest that IL4I1 could serve as a useful marker to identify high-risk patients and as a new target for future therapies. This research may help improve treatment for women with aggressive endometrial cancer and lead to more personalized and effective care.

## 1. Introduction

Endometrial cancer is one of the most common gynecological malignancies worldwide, with an increasing incidence in recent years [1]. The occurrence of this disease is closely related to various factors, including age, genetics, obesity, diabetes, and estrogen replacement therapy [2]. Current conventional diagnostic methods include ultrasound, MRI, and hysteroscopy [3,4], while molecular biology techniques such as genomic sequencing and expression profiling play a crucial role in tumor classification and prognosis evaluation. Endometrial cancer can be classified histologically into estrogen-dependent and estrogen-independent types, with the former having a better prognosis and the latter being associated with a poorer prognosis [5,6]. However, traditional histological classification is cumbersome, lacks consistency and stability, and does not offer targeted therapeutic options for certain subtypes, thus limiting its clinical application. In order to enhance the precision of patient prognosis assessments and inform clinical treatment strategies, the multi-omics study conducted by The Cancer Genome Atlas (TCGA) has introduced a molecular classification framework for endometrial cancer. This framework categorizes the disease into four distinct subtypes: POLE mutant, microsatellite instability (MMRd), low-copy number, and high-copy number. The high-copy number subtype is characterized by TP53 mutations and is associated with the worst prognosis [7]. With the development of molecular subtyping, the 2020 WHO classification of female reproductive system tumors further categorized endometrial cancer into four molecular subtypes: POLEmut, MMRd, P53abn, and NSMP. This molecular classification provides significant guidance for clinical prognosis of endometrial cancer [8]. Among these, POLE mutant endometrial cancer has the best prognosis, while P53 mutant endometrial cancer is considered to have the worst prognosis due to its higher malignancy, poor prognosis, and treatment complexity, which has attracted widespread attention [9].

IL4I1 (Interleukin 4-Induced Protein 1) is a key L-amino acid oxidase encoded by a gene located on human chromosome 19. It is primarily expressed in certain immune cells such as dendritic cells, macrophages, and B cells [10]. IL4I1 features a signal peptide sequence and an enzymatic activity region, which provide the basis for its catalytic reaction, and it may also be regulated by post-translational modifications such as phosphorylation and ubiquitination [11]. By catalyzing the deamination of specific amino acids, IL4I1 affects the metabolic state and immune response of cells [11]. Its activation and functional regulation involve complex signaling pathways, including the IL-4-induced JAK/STAT pathway, and possibly cross-regulation by factors such as IFN-γ and TNF-α [12,13]. In cancer development, IL4I1 regulates the tumor microenvironment, promotes immune evasion, and enhances tumor cell proliferation and metastasis, showing great potential as a therapeutic target [14]. While its roles in tumor immunity and metabolic reprogramming are increasingly recognized, it is important to note that IL4I1 also engages in a variety of non-oncogenic cellular functions. Acknowledging these broader physiological roles is essential for understanding its context-dependent behavior in cancer settings. Further research is needed to elucidate the precise mechanisms through which IL4I1 operates, which may ultimately offer novel strategies for cancer diagnosis and treatment.

In the field of cancer research, the widespread application of proteomics provides a new opportunity for precision medicine. By identifying biomarkers and molecular subtypes, proteomics promotes the exploration of personalized cancer diagnosis and treatment [15]. This study focuses on the expression of IL4I1 in tumors and its mechanism of affecting tumor progression through the regulation of the tumor microenvironment, especially its expression in P53 mutant endometrial cancer and its correlation with prognosis, aiming to provide a theoretical basis for future treatment strategies.

## 2. Materials and Methods

### 2.1. Patient Samples

This study initially screened 495 endometrial cancer patients treated at Shanghai First Maternity and Infant Hospital between 2010 and 2018. All patients provided written informed consent. Clinical information, including age, FIGO staging, tumor grade, histological type, depth of myometrial invasion, lymphovascular invasion, and lymph node metastasis, was retrieved from the hospital’s electronic information system.

### 2.2. Laser Capture Microdissection for Cancer Tissue Purification

Paraffin-embedded tissue samples were sectioned into multiple 10-μm thick slices. For smaller samples, the number of slices was increased as needed. The laser capture microdissection system was used, with tissue slices from the survival and death groups of P53 mutant endometrial cancer placed into collection tubes. Under the precise guidance of a pathologist, an appropriate magnification was selected to accurately target specific cells. After carefully delineating the selected target cells, adjustments were made to the laser aperture size, cutting speed, and laser power to ensure precise cutting and collection of the target cells into the designated tubes [16]. This meticulous process ensured high-quality sample preparation for subsequent analysis, providing a solid foundation for successfully identifying prognostic biomarkers for P53 mutant endometrial cancer.

### 2.3. Protein Extraction and Digestion

Samples were lysed and proteins extracted using SDT buffer (containing 4% SDS, 100 mM Tris-HCl, 1 mM DTT, pH 7.6). Protein concentration was determined using the BCA Protein Assay Kit (Bio-Rad, Hercules, CA, USA). Protein digestion followed Matthias Mann’s filter-aided sample preparation (FASP) technique. The resulting peptides were desalting treated using a C18 column (Empore™ SPE Cartridges C18, 3 ml, 7 mm i.d., Sigma, St. Louis, MO, USA), followed by concentration under vacuum conditions. Finally, the peptides were reconstituted in 40 μL of 0.1% formic acid solution for quantification using OD280 measurement.

### 2.4. LC-MS/MS Analysis

Samples were separated using the NanoElute HPLC system, equipped with a nano flow rate. Buffer A was a water solution containing 0.1% formic acid, and buffer B was a solution of 0.1% formic acid in 84% acetonitrile. The column was first equilibrated with 95% buffer A, followed by sample loading onto the loading column (Thermo Scientific Acclaim PepMap100, Waltham, MA, USA) using an automatic sample loading system. The samples were then separated through an analytical column (Thermo Scientific EASY column) at a flow rate of 300 nL/min. The separated samples were subsequently analyzed using the timsTOF Pro mass spectrometer in positive ion mode, with an ion source voltage set to 1.5 kV. MS and MS/MS data were collected via TOF for further analysis. The raw mass spectrometry data for each sample were integrated and analyzed using MaxQuant software (version 1.6.14) for database searching, identification, and quantification.

### 2.5. Bioinformatics Analysis

Bioinformatics analysis was performed using the ComplexHeatmap R package (compatible with R version 4.0.3) to carry out clustering analysis of the samples and protein expression levels in two dimensions and generate hierarchical clustering heatmaps. Differential protein expression analysis was conducted using the Bioconductor package in R Studio (version 4.0.3). Venn diagram analysis was performed to assess the overlap of identified proteins across different groups. The Limma R package (version 3.40.6) was used to identify differentially expressed genes (DEGs). For significant differential protein screening, a fold change (FC) >2.0 (upregulated more than 2.0-fold or downregulated less than 0.5-fold) and *p* < 0.05 were applied as the cutoff criteria to identify the upregulated and downregulated proteins between comparison groups. A bar plot was used to visualize these results. To visualize the significant differences in protein expression between comparison groups, a volcano plot was created, where significantly downregulated proteins were marked in blue (FC < 0.5 and *p* < 0.05), upregulated proteins in red (FC > 2.0 and *p* < 0.05), and non-differential proteins in gray. DEG with statistical significance were then subjected to Gene Ontology (GO) and Kyoto Encyclopedia of Genes and Genomes (KEGG) pathway analysis. GO terms were categorized into biological processes (BP), cellular components (CC), and molecular functions (MF). GO and KEGG enrichment results were visualized using ggplot2.

### 2.6. Immunohistochemistry

Formalin-fixed, paraffin-embedded (FFPE) uterine cancer tissue sections (4 μm) were subjected to immunohistochemical (IHC) staining. The specimens were deparaffinized and rehydrated, then incubated with the following primary antibodies: IL4I1 (1:1000 dilution, ab222102, Abcam, Waltham, MA, USA), p53 (1:100 dilution, ab32389, Abcam, USA), MLH1 (1:100 dilution, MAB-0838, Maixin, Tongliao, China), MSH2 (1:100 dilution, MAB-0836, Maixin, China), MSH6 (1:100 dilution, MAB-0831, Maixin, China), and PMS2 (1:100 dilution, RMA-0775, Maixin, China) (Table S1). After washing, the sections were incubated with biotin-conjugated secondary antibodies, followed by incubation with streptavidin-HRP. The sections were finally visualized using 3,3′-diaminobenzidine substrate. Images were captured using the Mantra system (PerkinElmer, Waltham, MA, USA) with the same exposure time for all slides. For the interpretation of MMR (mismatch repair) protein expression: if all four MMR proteins were normally expressed in the tumor cell nuclei, the sample was classified as pMMR; if one or more MMR proteins showed abnormal or absent expression in the tumor cell nuclei, it was classified as MMRd (Supplementary Figure S1). P53 protein expression was considered abnormal when it showed complete absence, strong diffuse positivity in the cell nucleus, or cytoplasmic expression, suggesting a TP53 gene mutation. Conversely, normal P53 expression was indicated by scattered positive expression in the cell nucleus, indicating the TP53 gene was likely wild-type [17,18,19] (Supplementary Figure S2).

### 2.7. POLE Mutation Analysis

Pathogenic mutations in the proofreading domain of POLE, specifically in exons 9-14, were assessed through Sanger sequencing [20]. The primer sequences used for amplification are provided in Table S2. PCR products were purified using the QIAquick PCR Purification Kit (Qiagen, Hilden, Germany) and directly sequenced using the ABI PRISM^®^ 3100-Avant Genetic Analyzer (Applied Biosystems, Waltham, MA, USA) and the SeqStudio Genetic Analyzer (Thermo Fisher Scientific, Waltham, MA, USA).

### 2.8. Western Blotting

Proteins were extracted using RIPA lysis buffer, and their concentrations were determined using the BCA assay. Approximately 20 µg of protein was loaded into each lane, separated by 10% SDS-PAGE, and transferred onto a PVDF membrane. The membrane was blocked with 5% non-fat milk at room temperature for 1 h, followed by overnight incubation at 4 °C with primary antibodies. The primary antibodies used were anti-IL4I1 (ab222102, Abcam) and anti-GAPDH (ab8245, Abcam). Afterward, the membrane was incubated with secondary antibodies at room temperature for 1 h. Western blots were developed using an ECL Western blotting substrate and quantified with ImageJ software (version 1.53).

### 2.9. IL4I1 Expression in Endometrial Carcinoma from TCGA

The TCGA Endometrial Carcinoma (EC) database includes data from 546 EC samples and 35 normal tissue specimens. The UALCAN portal (http://UALCAN.path.uab.edu (accessed on 16 October 2023)) was used for gene expression and survival analysis of IL4I1 in TCGA. The R limma package was applied to obtain IL4I1 expression data across various cancer types in the TCGA database. Subsequently, survival data was merged with the expression data, and a Wilcoxon rank-sum test was used to compare the data between the two groups.

### 2.10. Cell Culture and Lentiviral Infection

The human endometrial carcinoma cell lines HEC-1b and KLE, both identified as P53 mutant, were obtained from the Translational Medicine Center at the Obstetrics and Gynecology Hospital of Tongji University. These cell lines were cultured in DMEM medium (Hyclone, Beijing, China) supplemented with 10% fetal bovine serum (Gibco, Carlsbad, CA, USA) and maintained in a 37 °C incubator with 5% CO_2_. Penicillin (100 mg/L) was added to the medium for routine maintenance. IL4I1-knockout cell lines were generated using CRISPR/Cas9 technology. The CRISPR-Cas9 lentiviral vector (lentiCas9-Blast) was purchased from Genomeditech (Shanghai, China). The target IL4I1 sgRNA sequences used were as follows: sgRNA-1: CCCAACGATGACTTCTGTCC, sgRNA-2: ATGGCCTTTATGGTTAGCCC, and sgRNA-3: GTGCTGAGAGAGCCCCCCAG. CRISPR/Cas9-mediated transfection of IL4I1 was performed according to previously described protocols [21]. IL4I1 knockout efficiency was confirmed by Western blot analysis. Importantly, all cell-based functional experiments, including lentiviral knockout and phenotypic assays, were exclusively performed in TP53-mutant endometrial carcinoma cell lines (HEC-1b and KLE), with no normal endometrial epithelial cell lines used in this study. This ensures that all observed effects reflect cancer-specific mechanisms rather than physiological processes in non-malignant cells.

### 2.11. Cell Proliferation, Migration, and Invasion Assays

Cell proliferation, migration, and invasion assays were conducted as previously described [22]. For the cell proliferation assay, cells were cultured in groups and their optical density was measured at different time points using a CCK-8 reagent to plot the proliferation curve. In the migration assay, cells were serum-starved and then seeded in the upper chamber of a Transwell plate coated with diluted Matrigel. Cell suspensions were added to the upper chamber, while complete medium containing 10% FBS was added to the lower chamber. After 1–2 days of incubation, cells were fixed, stained, and examined using a microscope. The results were quantified using ImageJ software. For the invasion assay, Transwell chambers were pre-coated with 5% Matrigel (BioCoat, Guangzhou, China) in DMEM/F12 and incubated at 37 °C for 1 h. The procedure was then repeated as in the migration assay.

### 2.12. In Vivo Tumor Model

Nude mice (female, 4 weeks) were obtained from the Animal Experimentation of Shanghai First Maternity and Infant Hospital (Shanghai, China). HEC-1b and KLE cells were both identified as P53 mutant type and sourced from the Translational Medicine Center Laboratory of the Obstetrics and Gynecology Hospital of Tongji University. 1 × 10^7^ parental, L4I1 KO HEC-1B cells and KLE cells suspended in 100 μL PBS were subcutaneously injected into the subaxillary region of the nude mice to generate tumors with a size of 60 mm^3^. Sixteen mice were randomly divided into four groups (*n* = 4 per group). The tumor size was monitored every 2 days by measuring diameters using Vernier calipers, and the volume was calculated as V = πl^2^s/6, where l is the long side and s is the short side. Mice were euthanized using CO_2_ inhalation followed by cervical dislocation, in accordance with the AVMA Guidelines for the Euthanasia of Animals. All procedures were approved by the Animal Ethics Committee of Tongji University (No: TJBG11125102).

### 2.13. Single-Cell RNA-Seq Analysis

Single-cell RNA-seq data (UCEC_GSE139555) were downloaded from the Tumor Immune Single-cell Hub web service (http://tisch.comp-genomics.org/home/ (accessed on 6 January 2025)) [23]. The data were processed and analyzed using R software packages, MAESTRO (version 4.1.0) and Seurat (version 4.0.3). t-SNE was applied to perform cell clustering and sub-group analysis.

### 2.14. Data Analysis

For comparing categorical variables, the Chi-square test or Fisher’s exact test was used. Kaplan–Meier (KM) survival curves were generated, and the log-rank test was employed to assess survival differences between groups. Statistical analyses were performed using SPSS Statistics 25.0, R software (version 3.6.3), and GraphPad Prism software (version 9.2). All statistical tests were two-sided, and *p*  <  0.05 was considered statistically significant.

## 3. Results

### 3.1. Molecular Subtyping of Endometrial Cancer

We examined samples from 495 high-grade endometrial cancer patients (Table 1), with the majority of cases (82.2%) classified as endometrioid adenocarcinoma. The remaining 68 cases (17.8%) included non-endometrioid adenocarcinomas: 51 cases (10.3%) of serous carcinoma, 6 cases (1.2%) of clear cell carcinoma, 20 cases (4%) of carcinosarcoma, and 11 cases (2.2%) of other types. POLE gene exons 9–14 were sequenced using first-generation Sanger sequencing, and immunohistochemistry was used to assess mismatch repair (MMR) proteins and P53 protein status. Based on these results, we classified the endometrial cancers into four molecular subtypes: POLE mutation (POLE mut), mismatch repair deficiency (MMRd), p53 abnormal (P53abn), and no specific molecular profile (NSMP). Among the cases, 60 (12.1%) were classified as POLE mut, 147 (29.7%) as MMRd, 62 (12.5%) as P53abn, and 226 (45.7%) as NSMP. These proportions align with those reported in the guidelines, confirming the accuracy of our molecular classification [24].

### 3.2. Selection of FIGO Stage I P53 Mutant Endometrial Cancer Samples

In this study, we selected cases of FIGO Stage I P53 mutant endometrial cancer from 62 patients and conducted comprehensive follow-up to gather detailed prognostic information. As of 30 June 2022, we classified the patients into two groups based on their survival status: the survival group (*n* = 20) and the death group (*n* = 13) (Table 2). The survival group consisted of patients who were still alive at the end of the follow-up period and had no recurrence or metastasis at the last follow-up, while the death group included patients who died due to recurrence or metastasis during the follow-up.

### 3.3. Proteomic and Bioinformatics Analysis

**Figure 1:**
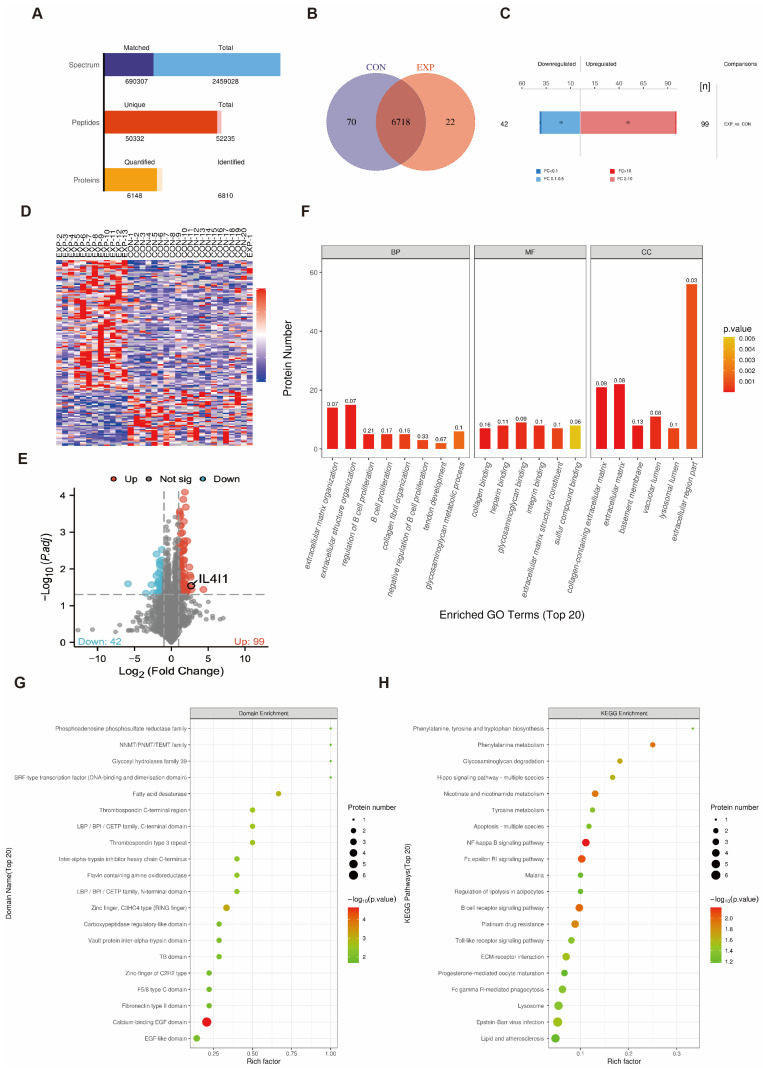
Proteomics Analysis and Functional Enrichment of Differentially Expressed Proteins. (**A**) Bar chart of identification and quantification results. (**B**) Venn diagram of identified proteins across all sample groups. (**C**) Bar chart of protein quantification differences between the survival and death groups. (**D**) Cluster analysis of differentially expressed proteins between the survival and death groups. (**E**) Volcano plot showing the significance and fold-change of proteins between control and experimental groups, with IL4I1 highlighted. (**F**) Top 20 enriched Gene Ontology (GO) terms for biological processes (BP), molecular functions (MF), and cellular components (CC) of differentially expressed proteins. (**G**) Domain enrichment analysis highlighting significant protein domains in the differentially expressed proteins. (**H**) KEGG pathway enrichment analysis for differentially expressed proteins, with significant pathways indicated.

The proteomic analysis identified a total of 52,235 peptides, of which 50,332 were unique peptides, leading to the identification of 6810 proteins, with 6148 proteins being quantified (Figure 1A). These results indicate a high spectral match rate and specificity, ensuring reliable data quality. Figure 1B illustrates the overlap of identified proteins across different sample groups.

In the differential expression analysis, the bar plot (Figure 1C) highlights the differences between the death group and the survival group, with 99 upregulated proteins and 42 downregulated proteins (fold change FC > 2, *p* < 0.05). The volcano plot (Figure 1E) visualizes the statistical significance and fold change of each protein, revealing that IL4I1 was significantly overexpressed in the death group compared to the survival group. To further classify differentially expressed proteins, we applied a hierarchical clustering algorithm, and the results were visualized using a heatmap (Figure 1D). This clustering analysis clearly distinguished between comparison groups based on protein expression patterns, demonstrating the dataset’s suitability for downstream analyses. In terms of functional enrichment analysis, Gene Ontology (GO) enrichment analysis revealed significant involvement of key biological processes such as extracellular matrix organization, extracellular structure organization, and B cell proliferation regulation, as well as molecular functions such as collagen binding and heparin binding (Figure 1F). To explore domain enrichment characteristics of differentially expressed proteins, we performed Fisher’s Exact Test, identifying significant enrichment in specific protein domains (Figure 1G). Kyoto Encyclopedia of Genes and Genomes (KEGG) pathway analysis highlighted significant alterations in NF-κB signaling, FcεRI signaling, and other critical pathways (Figure 1H). These findings provide valuable insights into the molecular mechanisms underlying biological processes and highlight potential biomarkers or therapeutic targets for further research.

### 3.4. IL4I1 Expression in Endometrial Cancer Tissue

**Figure 2:**
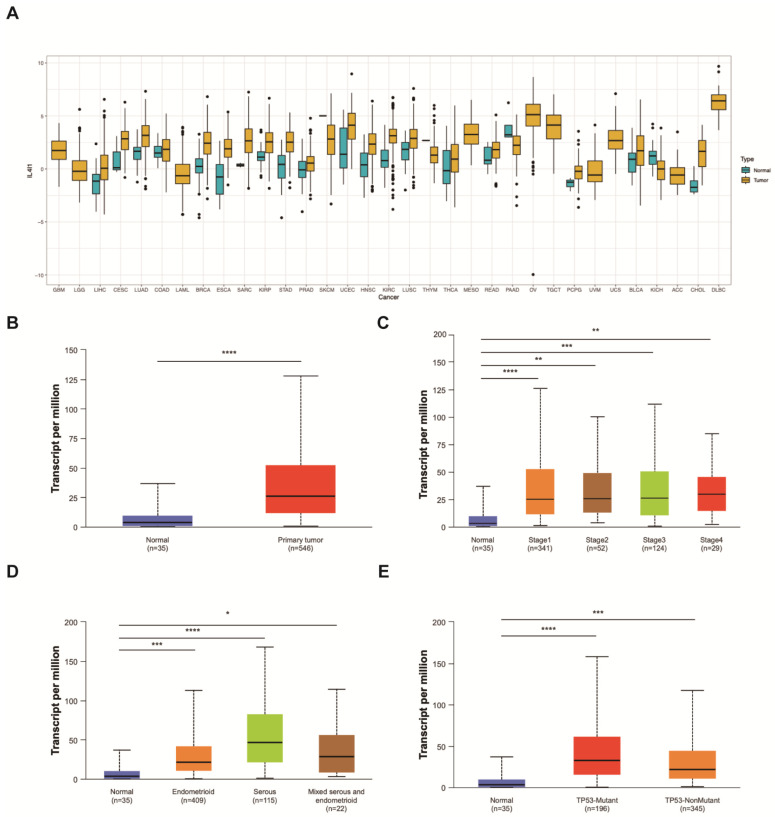
IL4I1 expression is significantly up-regulated in EC and is associated with the clinical stage and unfavorable prognosis of patients with EC. (**A**) The mRNA expression level of IL4I1 in TCGA pan-cancer. (**B**) IL4I1 mRNA expression in normal endometrium tissues and tumor tissues of EC from TCGA EC cohort (http://ualcan.path.uab.edu/ (accessed on 16 October 2023)). (**C**) IL4I1 mRNA expression in EC at different pathological stages from TCGA EC cohort. (**D**) IL4I1 mRNA expression in different histological subtypes of EC from TCGA EC cohort. (**E**) IL4I1 mRNA expression in different P53 expression statuses of EC from the TCGA EC cohort. * *p*  <  0.05, ** *p*  <  0.01, *** *p*  <  0.001, **** *p*  <  0.0001.

The expression levels of IL4I1 in cancer and adjacent normal tissues were analyzed using the Pan-cancer dataset from the TCGA database. As shown in Figure 2A, IL4I1 is highly expressed in the majority of malignant tumors. In endometrial cancer (EC), IL4I1 expression was significantly higher than that in normal endometrial tissues (Figure 2B). Further investigation demonstrated that IL4I1 expression correlates with histological subtypes and prognosis. Notably, serous endometrial carcinoma, which has the poorest prognosis, exhibited the highest IL4I1 expression, whereas endometrioid adenocarcinoma, associated with a better prognosis, showed only slightly elevated IL4I1 expression compared to normal endometrial tissues (Figure 2C).

Additionally, this pattern was consistent across different cancer stages (Figure 2D), indicating that IL4I1 expression increases progressively with disease advancement, suggesting a potential role in tumor progression. To further investigate its association with molecular subtypes, we compared IL4I1 expression between TP53-mutant and TP53-wild-type EC cases. As shown in Figure 2E, IL4I1 expression was significantly higher in TP53-mutant EC, further supporting its correlation with tumor aggressiveness and poor prognosis.

### 3.5. Differential Expression of IL4I1 in TP53-Mutant Endometrial Cancer

**Figure 3:**
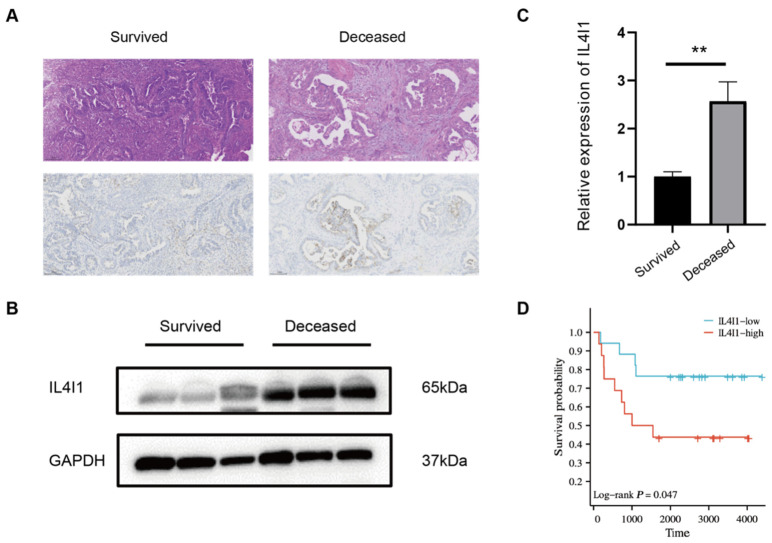
IL4I1 Expression and Prognostic Significance in TP53-Mutant Endometrial Cancer. (**A**) Expression of IL4I1 in cancer tissues from the death group and survival group; scale bar: 200 μm. (**B**,**C**) Western blot analysis of IL4I1 levels in cancer tissues from the death group and survival group. (**D**) Kaplan–Meier survival plots of OS according to IL4I1 mRNA expression in P53mut EC patients from our sample cohort. ** *p*  <  0.01. The original Western blot figures can be found in Supplementary File S1.

To explore IL4I1 expression differences in TP53-mutant EC, we performed immunohistochemical (IHC) analysis comparing IL4I1 levels between survival and death groups. Results indicated that IL4I1 was significantly upregulated in the death group (Figure 3A). Consistently, Western blot analysis further validated these findings, showing higher IL4I1 expression in the death group compared to the survival group (Figure 3B,C).

### 3.6. IL4I1 Expression and Prognostic Analysis in TP53-Mutant Endometrial Cancer

To assess the prognostic significance of IL4I1 expression, we stratified 33 TP53-mutant endometrial cancer (EC) patients into high- and low-expression groups based on the median IL4I1 expression level. Kaplan–Meier survival analysis revealed that patients with high IL4I1 expression had significantly lower overall survival (OS) compared to the low-expression group (Figure 3D). Log-rank test confirmed a statistically significant survival difference between the two groups (*p* = 0.047), indicating that high IL4I1 expression is associated with poor prognosis in TP53-mutant EC.

### 3.7. Generation of Stable IL4I1 Knockout HEC-1B and KLE Endometrial Cancer Cell Lines

**Figure 4:**
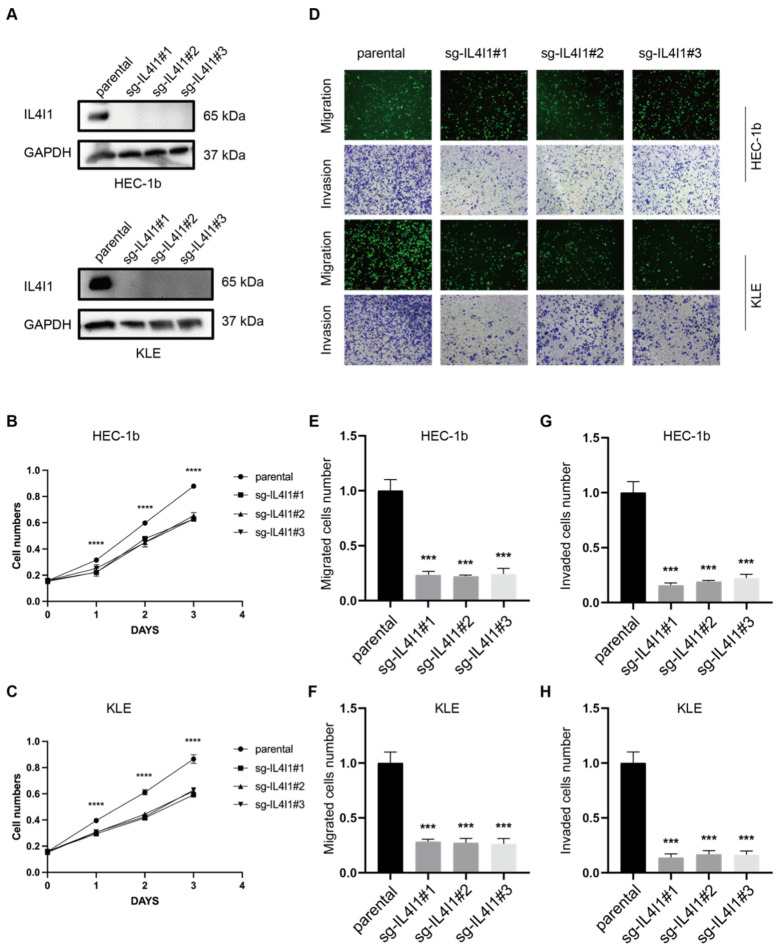
IL4I1 KO inhibits the proliferation, migration, and invasion capabilities of P53-mutant endometrial cancer cells. (**A**) Detection of IL4I1 knockout by Western blot. (**B**,**C**) CCK8 assay to assess changes in cell proliferation after IL4I1 knockout. (**D**–**H**) Transwell assay to assess changes in migration and invasion abilities of HEC-1b cells after IL4I1 knockout. *** *p*  <  0.001, **** *p*  <  0.0001. The original Western blot figures can be found in Supplementary File S1.

To investigate the functional role of IL4I1 in TP53-mutant EC progression, CRISPR/Cas9-mediated gene editing was employed to establish IL4I1-knockout (KO) HEC-1B and KLE cell lines. Western blot analysis confirmed the successful depletion of IL4I1 in the knockout cells (Figure 4A), as no detectable IL4I1 protein expression was observed in sg-IL4I1#1, sg-IL4I1#2, and sg-IL4I1#3 groups compared to the parental control.

### 3.8. IL4I1 Knockout Suppresses Cell Proliferation, Migration, and Invasion in TP53-Mutant Endometrial Cancer

**Figure 5:**
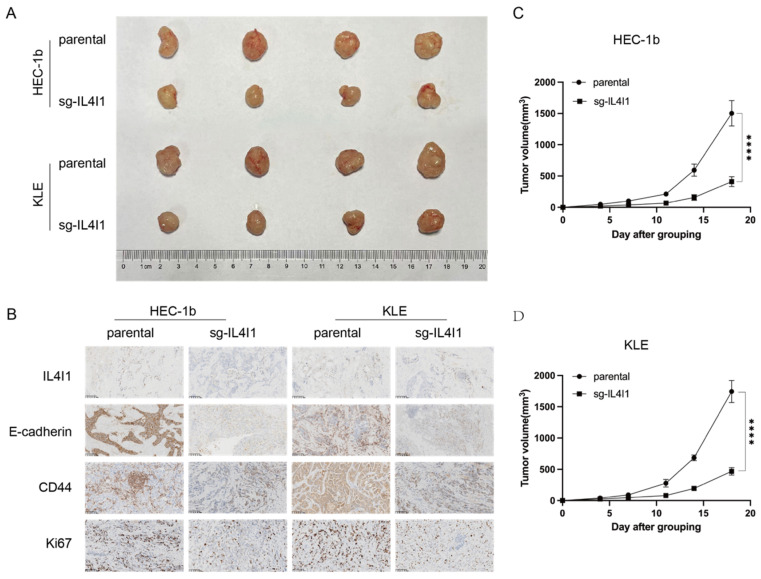
The role of IL4I1 in tumour growth and metastasis in a xenograft model. (**A**,**C**,**D**) Effects of IL4I1 knockout on tumour growth and growth curves (*n* = 4 per group; **** *p* < 0.0001). (**B**) Representative images of immunohistochemistry staining showing the expression of IL4I1, E-cadherin, CD44, and Ki67; scale bar: 200 μm.

To evaluate the effects of IL4I1 depletion on tumor cell behavior, CCK-8 proliferation assays were performed. Results showed that IL4I1 knockout significantly inhibited the proliferation of both HEC-1B and KLE cells (*p* < 0.0001, Figure 4B,C). Furthermore, Transwell migration and invasion assays demonstrated a marked reduction in the migratory and invasive abilities of IL4I1-KO cells compared to controls (*p* < 0.001, Figure 4D–H). In in vivo xenograft models, subcutaneous implantation of IL4I1-KO HEC-1B and KLE cells into nude mice resulted in significantly reduced tumor growth compared to parental cells (Figure 5A,C,D). Additionally, immunohistochemical (IHC) analysis revealed a decrease in IL4I1, E-cadherin, CD44, and Ki67 levels in tumors derived from IL4I1-KO cells (Figure 5B).

Collectively, these findings suggest that IL4I1 plays a critical role in promoting tumor aggressiveness in TP53-mutant EC, highlighting its potential as a therapeutic target for this highly invasive cancer subtype.

### 3.9. IL4I1 Expression Profiling and Its Potential Role in the Tumor Microenvironment

**Figure 6:**
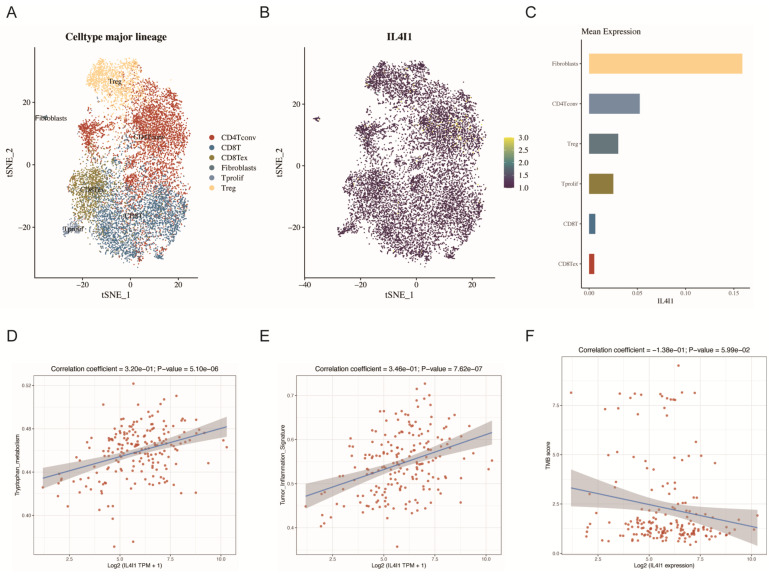
The Expression of IL4I1 in the Tumor Microenvironment. (**A**) t-SNE plot of major cell lineages in the tumor microenvironment. (**B**) t-SNE plot depicting the expression of IL4I1 across different cell types. (**C**) Bar graph showing the mean expression of IL4I1 across major cell types. (**D**) Correlation analysis of IL4I1 expression with tryptophan metabolism. (**E**) Correlation of IL4I1 expression with tumor-inflammation signature. (**F**) Correlation of IL4I1 expression with TMB (tumor mutational burden).

Based on the single-cell RNA-seq data from GSE139555, we investigated the expression profile of IL4I1 within the endometrial cancer (EC) microenvironment. Our analysis revealed that IL4I1 is predominantly expressed in fibroblasts, with minimal expression observed in CD8+ exhausted T cells (CD8Tex), suggesting that IL4I1 may play a role in tumor immune suppression (Figure 6A–C).

Further correlation analysis using the TCGA database on TP53-mutant EC samples revealed that IL4I1 expression was significantly positively correlated with tryptophan metabolism and tumor inflammation features, while exhibiting a negative correlation with tumor mutational burden (TMB) (Figure 6D–F). These findings suggest that IL4I1 may influence the immune microenvironment of EC through metabolic remodeling and inflammatory modulation, which could potentially promote tumor progression and immune evasion, pending further experimental validation.

## 4. Discussion

The role of IL4I1 in tumor progression has become increasingly complex. Its expression in the tumor microenvironment is considered an integral part of the immune evasion mechanism. Overexpression of IL4I1 can promote tryptophan catabolism [25], which suppresses the activity of T cells and NK cells, thereby helping tumor cells evade immune surveillance [26,27]. Additionally, IL4I1 may promote tumor growth and metastasis by modulating immune cell functions. These observations have sparked significant research interest in IL4I1 as a potential therapeutic target.

The expression of IL4I1 across various tumor types reveals a complex relationship with disease prognosis. In cancers such as triple-negative breast cancer, ovarian cancer, clear cell renal carcinoma, glioblastoma, and low-grade lymphomas, IL4I1 expression levels correlate closely with tumor progression, clinical-pathological features, malignancy, and patient survival, making it a promising prognostic biomarker for various cancers [28,29,30,31,32]. A pan-cancer analysis through TCGA revealed that IL4I1 is involved in multiple cellular functions and signaling pathways. For example, gene ontology (GO) annotations indicated that IL4I1 is associated with leukocyte migration and cell adhesion, both critical processes in tumor inflammation and immune responses [33,34]. Furthermore, KEGG pathway analysis identified IL4I1 as a key regulator of several biological processes, including chemokine signaling, cytokine receptor interactions, and the JAK-STAT pathway. However, in some tumors, such as follicular lymphoma, increased expression of IL4I1 may be associated with effective immune responses to tumors, reflecting a better prognosis. This dual role of IL4I1 underscores its complex regulatory mechanism in tumor development.

Endometrial cancer, one of the most common malignancies in women globally, has seen a rising incidence in recent years. While imaging technologies and molecular diagnostic techniques have been applied clinically, traditional therapeutic strategies are still limited for certain poor-prognosis subtypes of endometrial cancer. Particularly, P53-mutant endometrial cancer remains challenging due to its aggressive nature and poor prognosis, highlighting the urgent need for new therapeutic targets and prognostic biomarkers.

Based on our findings, IL4I1 is highly expressed in P53-mutant endometrial cancer and is closely linked to poorer prognosis. While this correlation does not establish causality, it supports the hypothesis that IL4I1 may contribute to tumor progression. Functional experiments demonstrated that IL4I1 knockout significantly inhibited the proliferation, migration, and invasion of P53-mutant endometrial cancer cells, suggesting a tumor-promoting role in this context. These findings are further supported by xenograft models, which confirmed the suppressive effects of IL4I1 loss in vivo. However, we acknowledge that these data reflect phenotypic consequences rather than direct molecular mechanisms, and that IL4I1 likely operates as part of a broader regulatory network. Future studies are warranted to delineate the specific pathways through which IL4I1 exerts its effects.

Taken together, our study contributes to the growing evidence that IL4I1 may modulate the tumor microenvironment and influence cancer aggressiveness. Given the importance of immune evasion and inflammation in endometrial cancer metastasis [35,36], IL4I1 may represent a novel candidate for further exploration as a therapeutic target. However, we emphasize that the molecular interactions remain to be elucidated, and the current findings should be viewed as a foundation for future mechanistic studies.

## 5. Conclusions

In summary, this study identifies IL4I1 as a significantly upregulated protein in TP53-mutant endometrial cancer and demonstrates its association with poor prognosis. Functional assays confirmed that IL4I1 promotes tumor cell proliferation, migration, and invasion, and is correlated with an immunosuppressive tumor microenvironment. While mechanistic pathways remain to be fully elucidated, our findings suggest that IL4I1 may serve as a candidate prognostic biomarker and a potential therapeutic target in this aggressive EC subtype. Future studies involving pathway-specific interventions and interaction mapping are needed to clarify IL4I1’s mechanistic role and assess its translational utility in precision oncology.

## Figures and Tables

**Table 1:** Molecular Subtypes and Clinical Pathological Characteristics of 495 Endometrial Cancer Patients.

Clinical Information		*n* (%)
Age	≤60	353 (71.3)
	>60	142 (28.7)
FIGO	I	400 (80.8)
	II	42 (8.5)
	III	47 (9.5)
	IV	6 (1.2)
Tumor Grade	Low grade	415 (83.8)
	High grade	80 (16.2)
Histological Type	Endometrioid adenocarcinoma	407 (82.2)
	Serous carcinoma	51 (10.3)
	Clear cell carcinoma	6 (1.2)
	Carcinosarcoma	20 (4.0)
	Others	11 (2.2)
Myometrial Invasion Depth	<1/2	374 (75.6)
	≥1/2	121 (24.4)
Lymph Node Metastasis	No	462 (93.3)
	Yes	33 (6.7)
Molecular Subtype	POLE mutation	60 (12.1)
	MMRd	147 (29.7)
	NSMP	226 (45.7)
	P53 mutation	62 (12.5)
LVSI	No	442 (89.3)
	Yes	53 (10.7)

**Table 2:** 33 cases of FIGO stage I P53 mutant endometrial cancer patients.

Clinical Information		Death *n* (%)	Survival *n* (%)	χ^2^	*p*
Age	<60	2 (15.4)	6 (30)	0.293	0.588
	≥60	11 (84.6)	14 (70)		
Myometrial Invasion Depth	<50%	7 (53.8)	16 (80)	1.464	0.226
	≥50%	6 (46.2)	4 (20)		
LVSI	No	12 (92.3)	15 (75)	0.636	0.425
	Yes	1 (7.7)	5 (25)		

## Data Availability

The datasets used and/or analyzed during the present study are available from the corresponding author upon reasonable request.

## Supplementary Materials

The following supporting information can be downloaded at: https://www.mdpi.com/article/10.3390/cancers17182986/s1, File S1: The original Western blot figures; Figure S1: Interpretation of MMR Protein Expression Results; Figure S2: Interpretation of P53 Protein Expression Results; Table S1: Antibodies used herein; Table S2: The Primer Sequences for Detection of POLE Exon 9-14 in the DNA Exonuclease Domain.
