# Supplementary material for: Proteomic Identification of IL4I1 as a Therapeutic Target in P53-Mutant Endometrial Cancer
Source: Cancers (Basel). 2025 Sep 12;17(18):2986. doi: 10.3390/cancers17182986 (PMC12468537; doi:10.3390/cancers17182986)
Supplement: Supplementary file 1 [file cancers-17-02986-s001.zip › File S1. The original Western blot figures.pptx]

## Slide 1
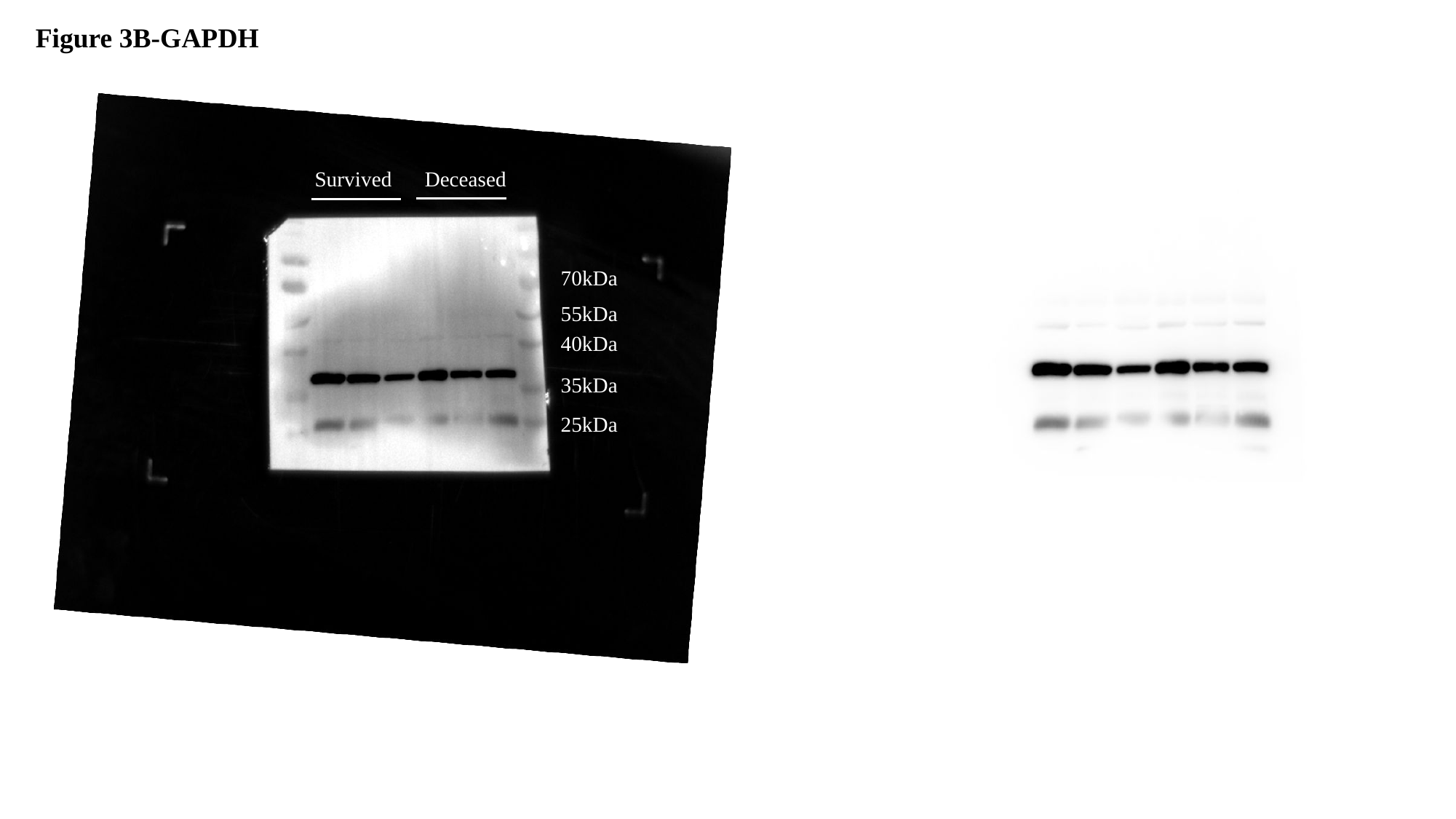

Figure 3B-GAPDH
Survived
Deceased
70kDa
55kDa
40kDa
35kDa
25kDa

## Slide 2
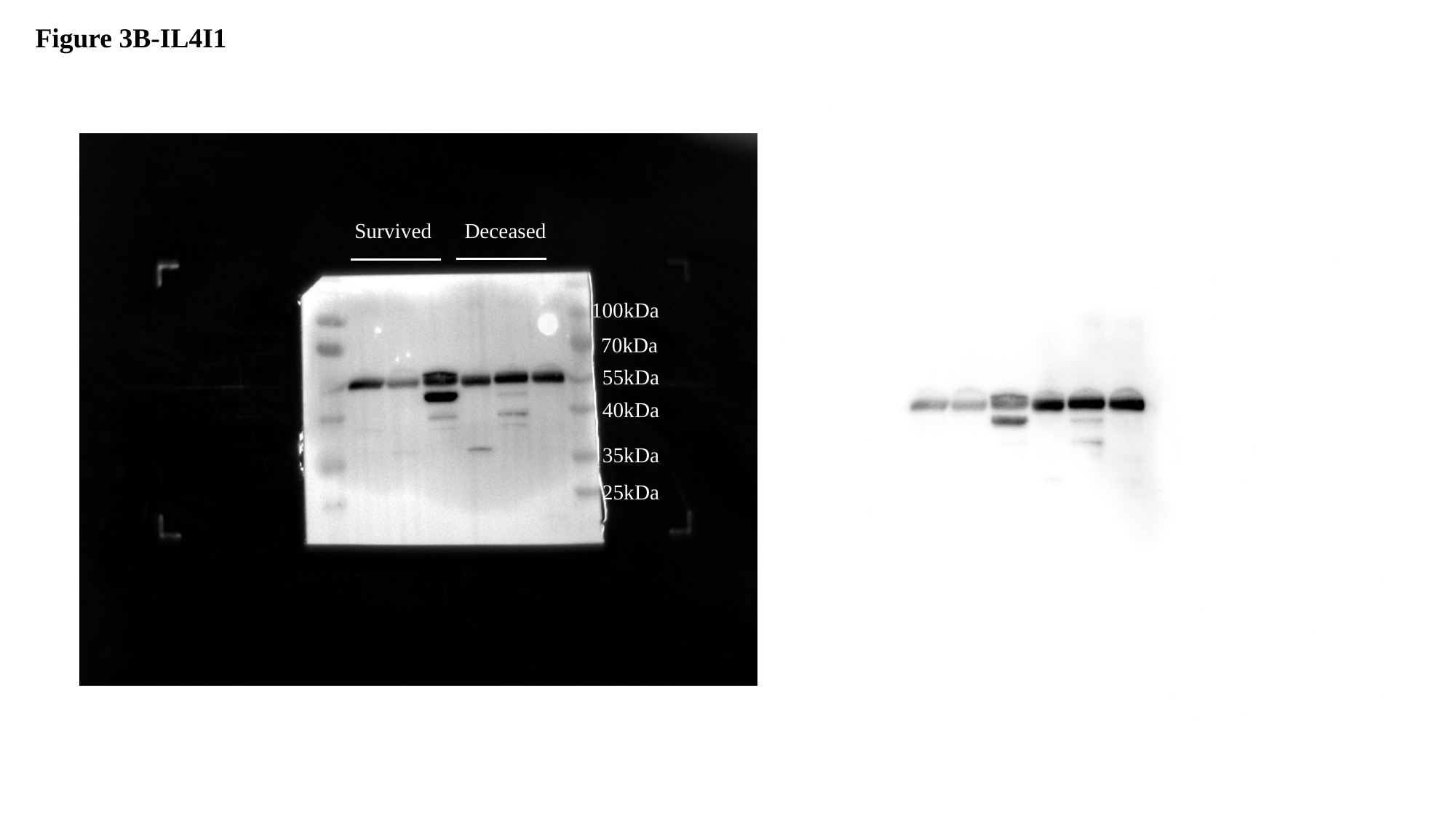

Figure 3B-IL4I1
Survived
Deceased
100kDa
70kDa
55kDa
40kDa
35kDa
25kDa

## Slide 3
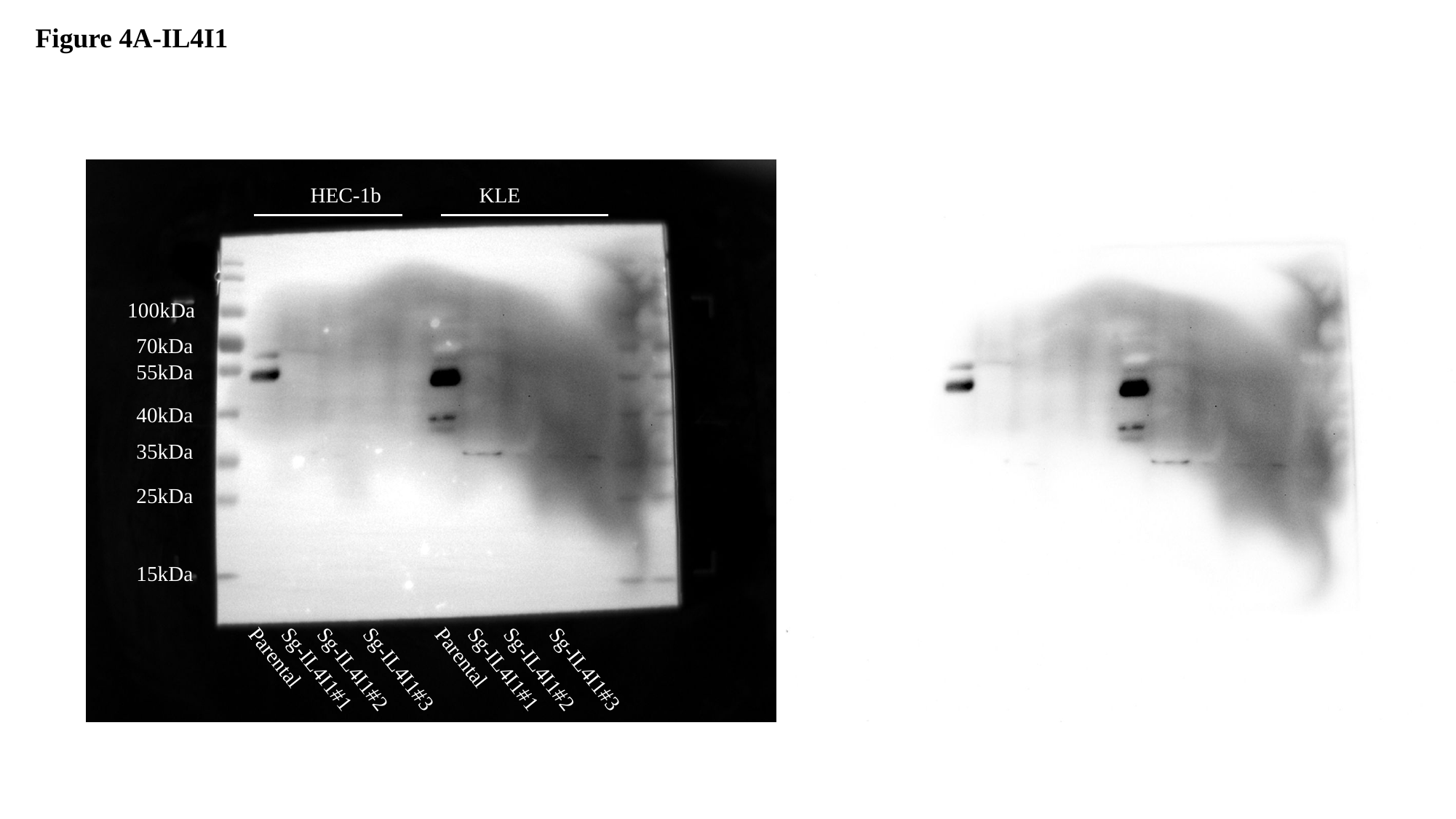

Figure 4A-IL4I1
HEC-1b
KLE
100kDa
70kDa
55kDa
40kDa
35kDa
25kDa
15kDa
Parental
Parental
Sg-IL4I1#1
Sg-IL4I1#2
Sg-IL4I1#3
Sg-IL4I1#1
Sg-IL4I1#2
Sg-IL4I1#3

## Slide 4
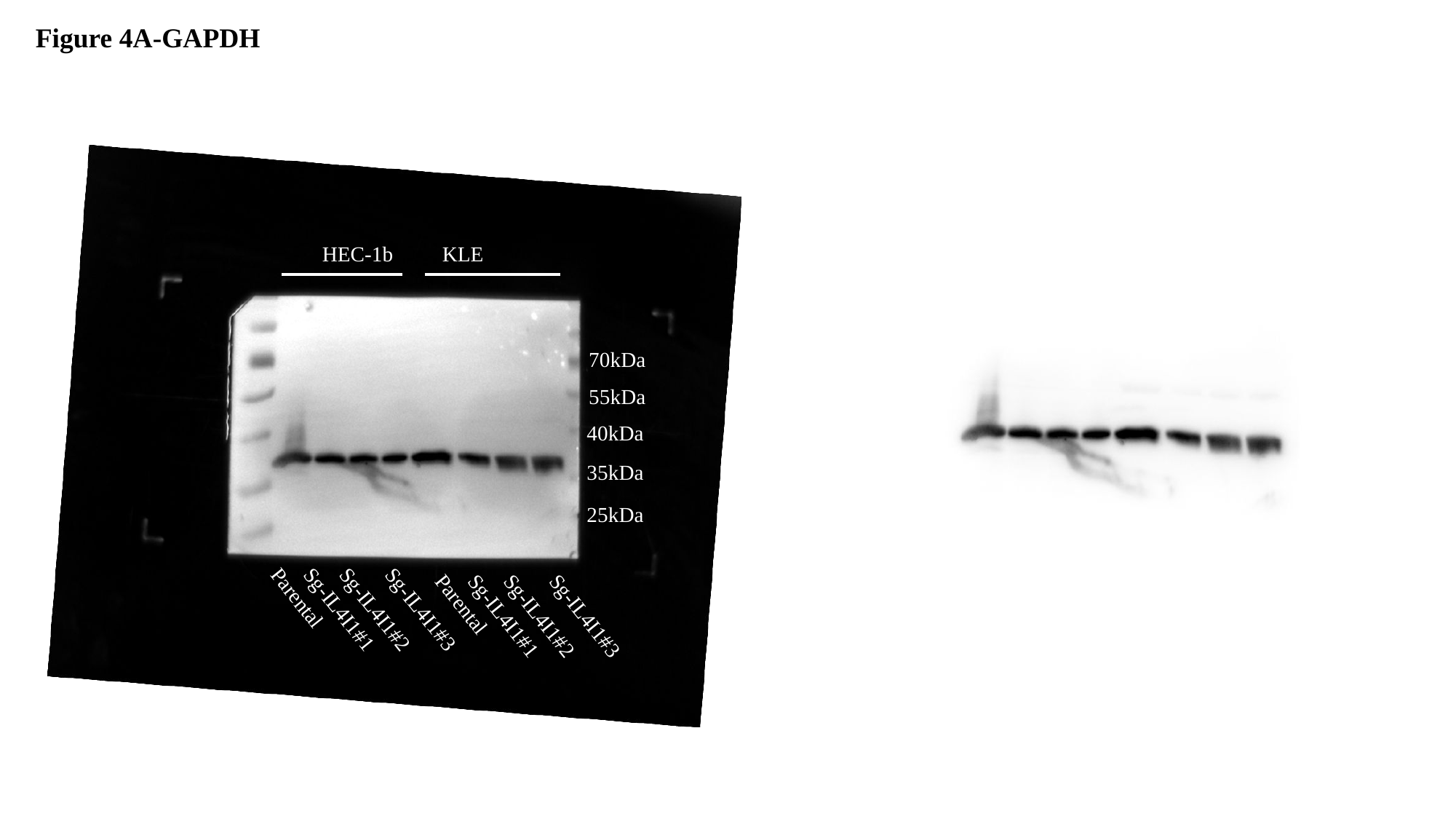

Figure 4A-GAPDH
HEC-1b
KLE
70kDa
55kDa
40kDa
35kDa
25kDa
Parental
Parental
Sg-IL4I1#1
Sg-IL4I1#2
Sg-IL4I1#3
Sg-IL4I1#1
Sg-IL4I1#2
Sg-IL4I1#3
