# Supplementary material for: Proteomic Identification of IL4I1 as a Therapeutic Target in P53-Mutant Endometrial Cancer
Source: Cancers (Basel). 2025 Sep 12;17(18):2986. doi: 10.3390/cancers17182986 (PMC12468537; doi:10.3390/cancers17182986)
Supplement: Supplementary file 1 [file cancers-17-02986-s001.zip › Supplemental materials.pdf]

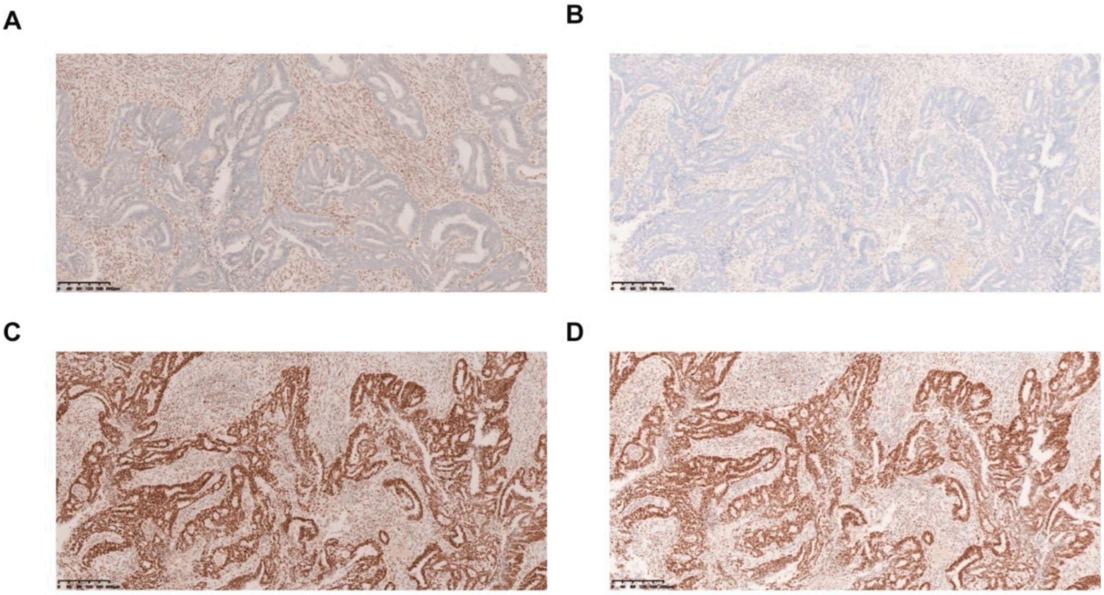

Supplementary Figure S1. Interpretation of MMR Protein Expression Results.

A. Loss of MLH1 Expression; B. Loss of PMS2 Expression; C. Normal Expression of MSH2; D. Normal Expression of MSH6

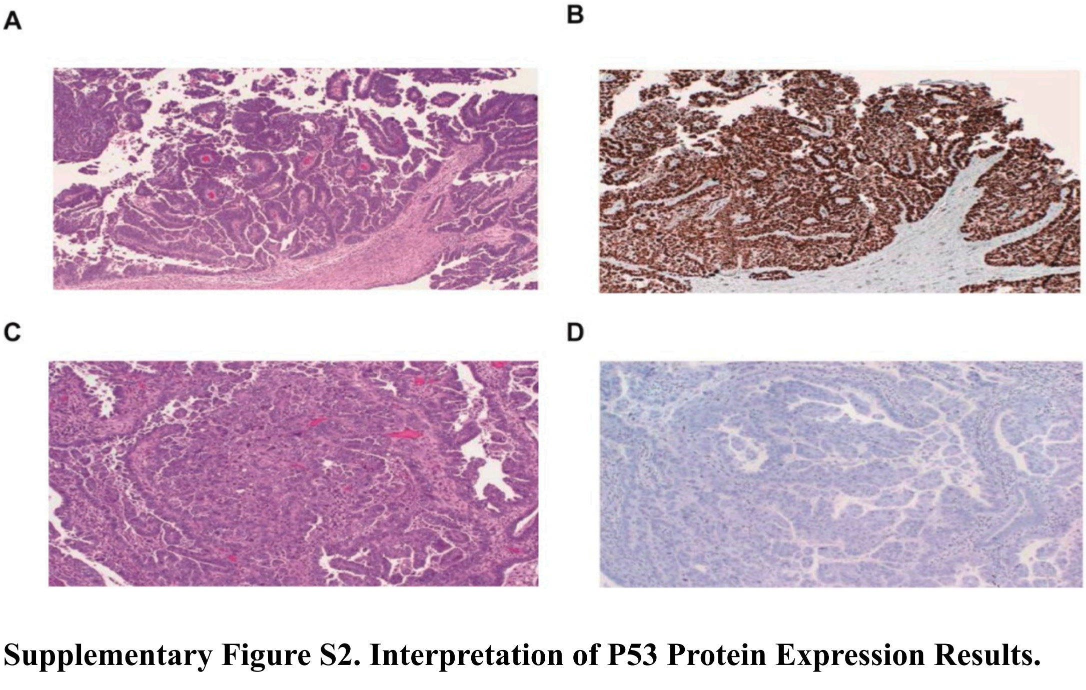

Supplementary Figure S2. Interpretation of P53 Protein Expression Results.

A, C. HE Staining; B. P53 Missense Mutation; D. P53 Nonsense Mutation

Supplementary Tables captions

Supplementary Table S1. Antibodies used herein.

Supplementary Table S2. The Primer Sequences for Detection of POLE Exon 9-14 in the DNA Exonuclease Domain
